# A Rare Case of Heterotopic Twin Pregnancy After Spontaneous Conception

**DOI:** 10.7759/cureus.78928

**Published:** 2025-02-13

**Authors:** Elitsa Gyokova, Yoana Kostadinova, Elizabeth A Odumosu

**Affiliations:** 1 Obstetrics and Gynecology, University Hospital Saint Marina - Pleven, Pleven, BGR; 2 Obstetrics and Gynecology, Medical University - Pleven, Pleven, BGR; 3 Obstetrics and Gynecology, University Hospital "Georgi Stranski", Pleven, BGR; 4 Faculty of Medicine, Medical University - Pleven, Pleven, BGR

**Keywords:** differential diagnosis, ectopic pregnancy, heterotopic pregnancy, intrauterine pregnancy, laparoscopy

## Abstract

Heterotopic pregnancy is a rare condition characterized by a simultaneous presence of intrauterine pregnancy and extrauterine (ectopic) pregnancy. Due to its rarity, reports in medical literature are limited. Various risk variables have been identified, and assisted reproductive techniques have been considered the most important risk factor.

A 37-year-old female, G8P5 (vaginal deliveries), after spontaneous conception, presented when her last menstrual period was five weeks and two days ago with abdominal pain. An initial ultrasound revealed an intrauterine gestational sac with a normal developing embryo. She was prescribed spasmolytics for pain and discharged. Despite treatment, the pain continued, and three weeks later the patient returned with severe abdominal pain. A repeat ultrasound confirmed a normally developing intrauterine pregnancy but also showed free fluid in the Douglas pouch and lower abdomen, raising suspicion for hemoperitoneum with unknown etiology. A diagnostic laparoscopy confirmed a rupture in the right tubal wall, likely due to a ruptured ectopic pregnancy. A salpingectomy was needed, and the intrauterine pregnancy was maintained undisturbed. The pregnancy progressed normally, and she delivered vaginally at 38 weeks.

The rarity of this condition and the presence of a normal intrauterine pregnancy often delay diagnosis. Early detection is essential in preventing complications, such as tubal rupture and intraabdominal hemorrhage. It is essential to underscore that heterotopic pregnancies may occur following spontaneous pregnancies. Additionally, in patients diagnosed with intrauterine pregnancies who exhibit abdominal pain or vaginal bleeding, it is crucial to exclude the possibility of extrauterine pregnancy, including those resulting from spontaneous conception.

Heterotopic pregnancy should be considered as a differential diagnosis in patients presenting with abdominal pain. The presence of an intrauterine gestational sac does not exclude the possibility of an ectopic pregnancy, and thorough examinations should be routinely performed.

## Introduction

Heterotopic pregnancy refers to the concurrent existence of an intrauterine and an extrauterine pregnancy. Although rare, this condition can escalate into a life-threatening situation if not promptly diagnosed and managed. Duverney first diagnosed it in the year 1708 during an autopsy [1]. Since then, it has been reported to occur in one in 300,000 naturally conceived pregnancies, and the incidence has increased from one in 100 to one in 1,500 pregnancies conceived using artificial reproductive techniques (ART) [2,3].

The exact etiology of heterotopic pregnancies remains unclear; however, certain risk factors have been identified. These include conception through ART, treatment for infertility, and a history of pelvic inflammatory disease (PID) [4]. Research indicates that ovarian hyperstimulation syndrome and numerous embryo transfers during IVF correlate with a heightened risk of heterotopic pregnancy. Tubal anomalies, including pelvic inflammatory illness and prior tubal or abdominopelvic surgery, have been recognized as risk factors [5]. Recognizing these risk factors can aid in identifying women at higher risk of developing heterotopic pregnancies, guiding further diagnostic testing in these cases. However, it is important to note that heterotopic pregnancies can still occur in women without these risk factors, underscoring the need for vigilance in all patients.

The extrauterine pregnancy, also referred to as an ectopic pregnancy, is not viable due to its abnormal implantation site. The most common location for an ectopic pregnancy is the fallopian tube, accounting for over 90% of cases [6]. The remaining 10% occur in other locations, such as the ovaries, cervix, abdomen, or cesarean scars. The growth of an ectopic pregnancy can lead to the rupture of surrounding structures, causing internal hemorrhage and potentially fatal complications. For example, a ruptured fallopian tube from a tubal ectopic pregnancy can result in severe bleeding and the loss of the affected tube.

Studying heterotopic pregnancies is critical for improving the diagnosis, as these cases pose significant diagnostic challenges. The simultaneous presence of a normal intrauterine pregnancy often diminishes suspicion of an ectopic pregnancy, potentially delaying further investigation [7]. Additionally, the overlap between symptoms of a normal early pregnancy - such as abdominal pain and vaginal bleeding - and those of heterotopic pregnancies further complicates diagnosis. Notably, approximately one in four women experience vaginal bleeding during early pregnancy and still go on to have a normal pregnancy [8]. With the increasing use of ART, the incidence of heterotopic pregnancies is expected to rise, reinforcing the importance of heightened clinical awareness and early detection. Early recognition and diagnosis of heterotopic pregnancies are crucial to prevent and mitigate these complications. If an ectopic pregnancy is missed - particularly in cases where only the intrauterine pregnancy is identified - the risk of life-threatening complications increases, endangering the mother's life and the viability of the intrauterine pregnancy.

The purpose of this research paper is to raise awareness among physicians about heterotopic pregnancy to facilitate earlier diagnosis, thereby reducing maternal and intrauterine fetal mortality. Additionally, it aims to explore available treatment options and analyze outcomes for both the mother and fetus.

## Case presentation

**Figure 1:**
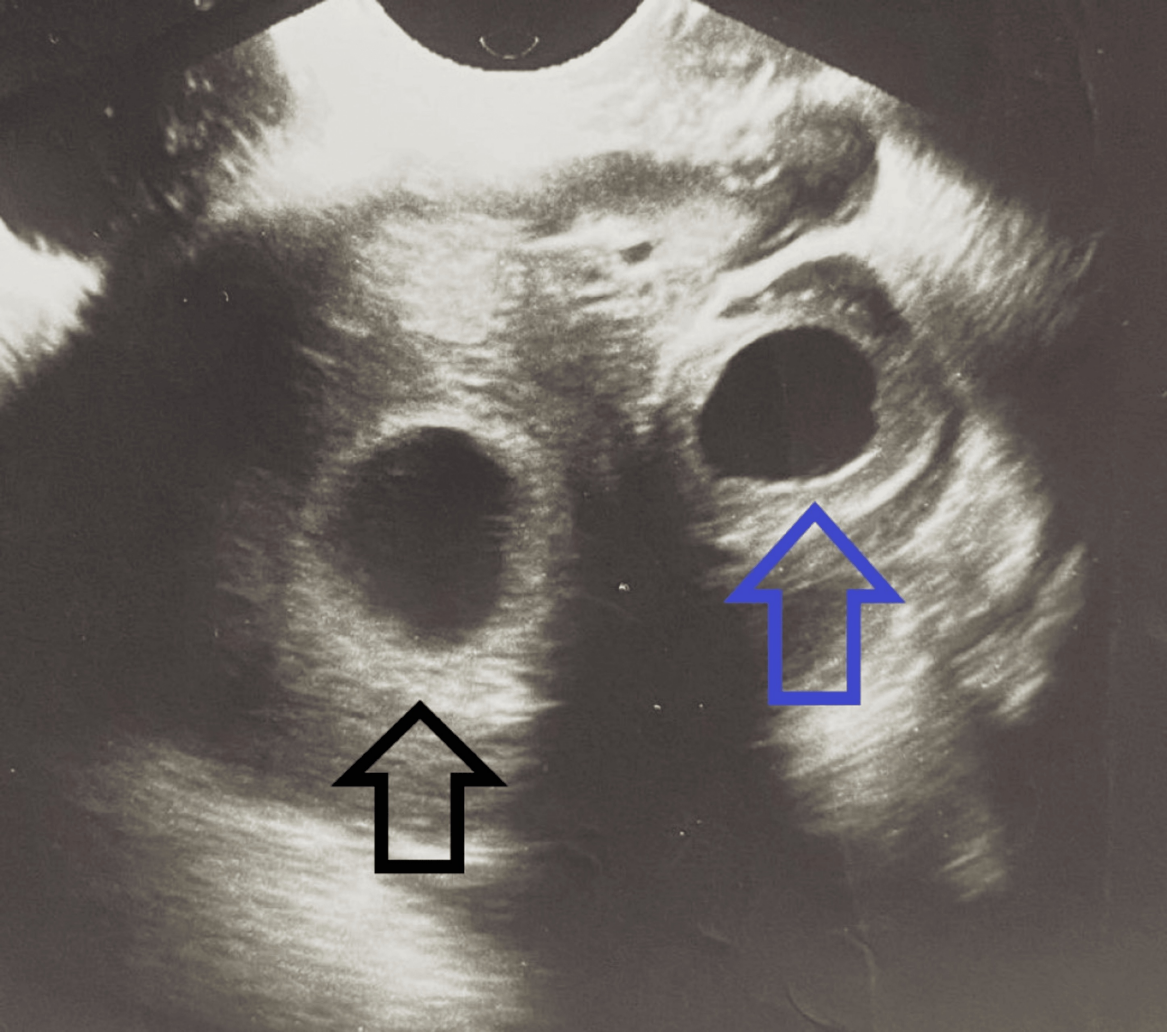
Normal intrauterine gestational sac and fluid-filled sac in the fallopian tube The black arrow points to the normal intrauterine gestational sac. The blue arrow points to the fluid-filled sac in the fallopian tube.

A 37-year-old female patient, gravida 8, para 5 (she has a history of five deliveries via vaginal birth and three terminations per personal reasons), presented to the Obstetrics department when her last menstrual period was five weeks and two days ago, following a spontaneous conception. The patient had no other significant gynecological history or surgeries. Her presenting vital signs were unremarkable: blood pressure 110/ 66 mmHg, pulse 68 beats/ minute, temperature 36.3 C). She possessed no prior history of infertility treatments, no history of contraceptive pills (either emergency or routine), or pelvic inflammatory illness, all of which are recognized risk factors for heterotopic pregnancy. The patient presented experiencing lower abdomen pain without vaginal bleeding or discharge for the last two days. A transvaginal ultrasound was performed to evaluate her pregnancy status and identify any other potential causes of her symptoms. The ultrasound revealed an intrauterine gestational sac (measuring 7.0 mm x 6.5 mm) with a normally developing embryo with a crown-rump length of 1.9 mm, confirming a viable pregnancy of five weeks and two days. Additionally, a fluid-filled sac with a double-decidual ring with measurements of 6.8 mm x 3.1 mm was observed in the fallopian tube, which was initially thought to be a cyst (Figure 1). The patient was prescribed drotaverine 40 milligrams and papaverine 50 milligrams three times daily as spasmolytics for pain management, discharged from the clinic, and advised to return if she experienced any further symptoms. No further testing, including serum levels of beta-human chorionic gonadotropin (b-hCG), was conducted at the attending physician's discretion.

**Figure 2:**
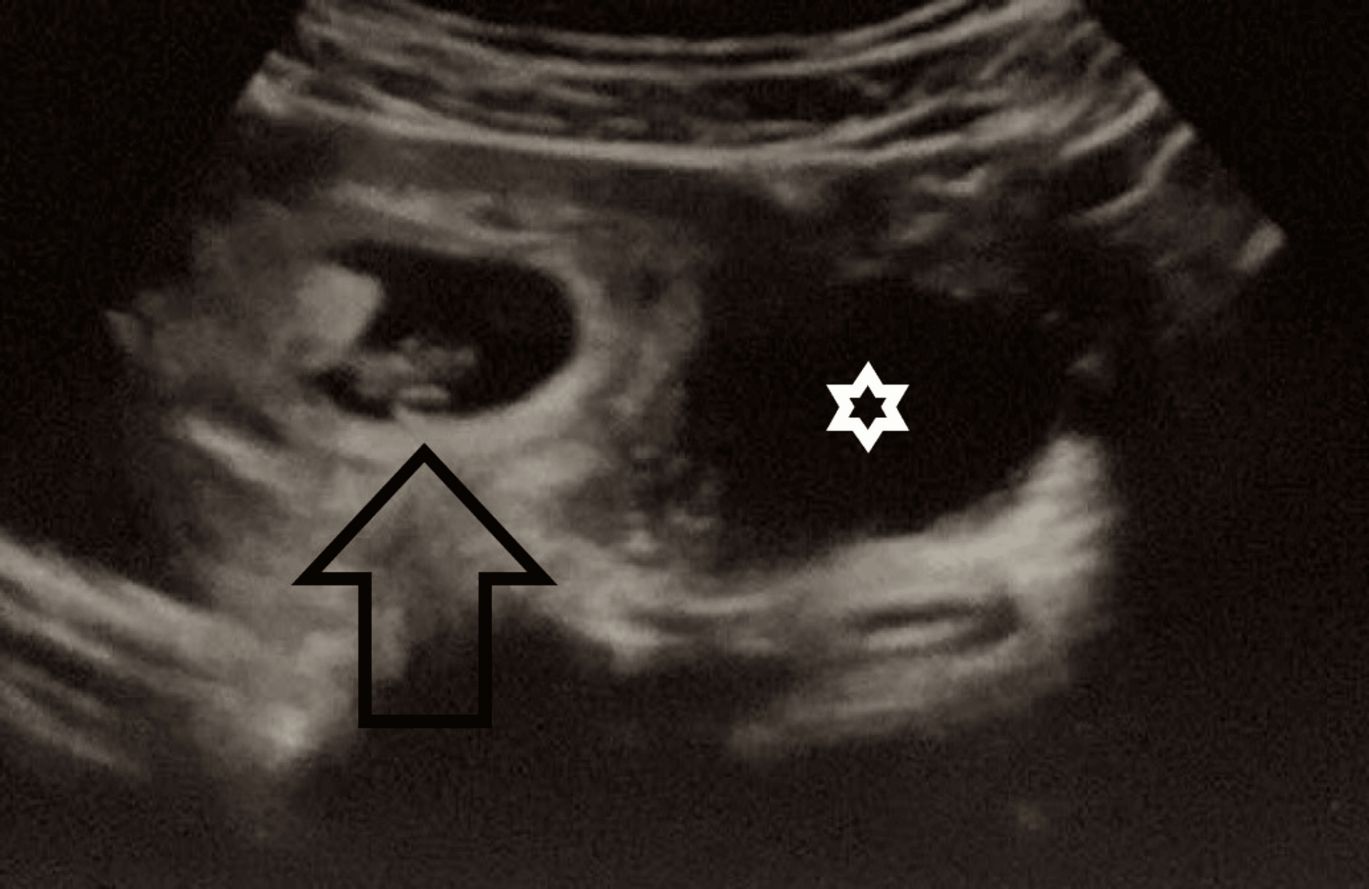
Ultrasound showing a normal developing intrauterine pregnancy with free-floating abdominal fluid in the Douglas pouch and lower abdomen. The black arrow points to the normal gestation sac in the uterine cavity. The white star presents the free-floating fluid in the Douglas pouch.

Despite the initial treatment, the patient’s pain persisted. Two weeks later, at seven weeks and six days gestation, she returned with intense sharp abdominal pain and indications of an acute abdomen, characterized by rebound soreness and diminished bowel sounds. A repeat ultrasound confirmed a normally developing intrauterine pregnancy described by an intrauterine gestational sac measuring 27.5 mm x 31.0 mm with embryo, measuring 14.5 mm crown-rump length (equal for seven weeks and six days) and revealed free floating abdominal fluid in the Douglas pouch and lower abdomen, raising suspicion of hemoperitoneum with an unknown etiology (Figure 2).

**Figure 3:**
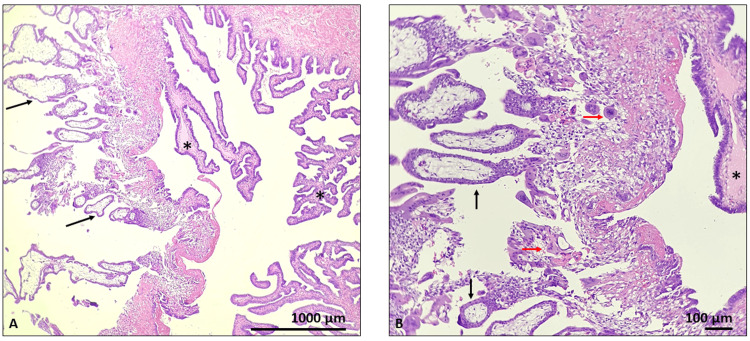
Histological specimen of the fallopian tube Hematoxylin and eosin staining: visible intraluminal chorionic villi (black arrow), trophoblasts within fibrin (red arrow), mucosal folds with tubal epithelia (black stars); magnification x40 (A) and x100 (B)

**Figure 4:**
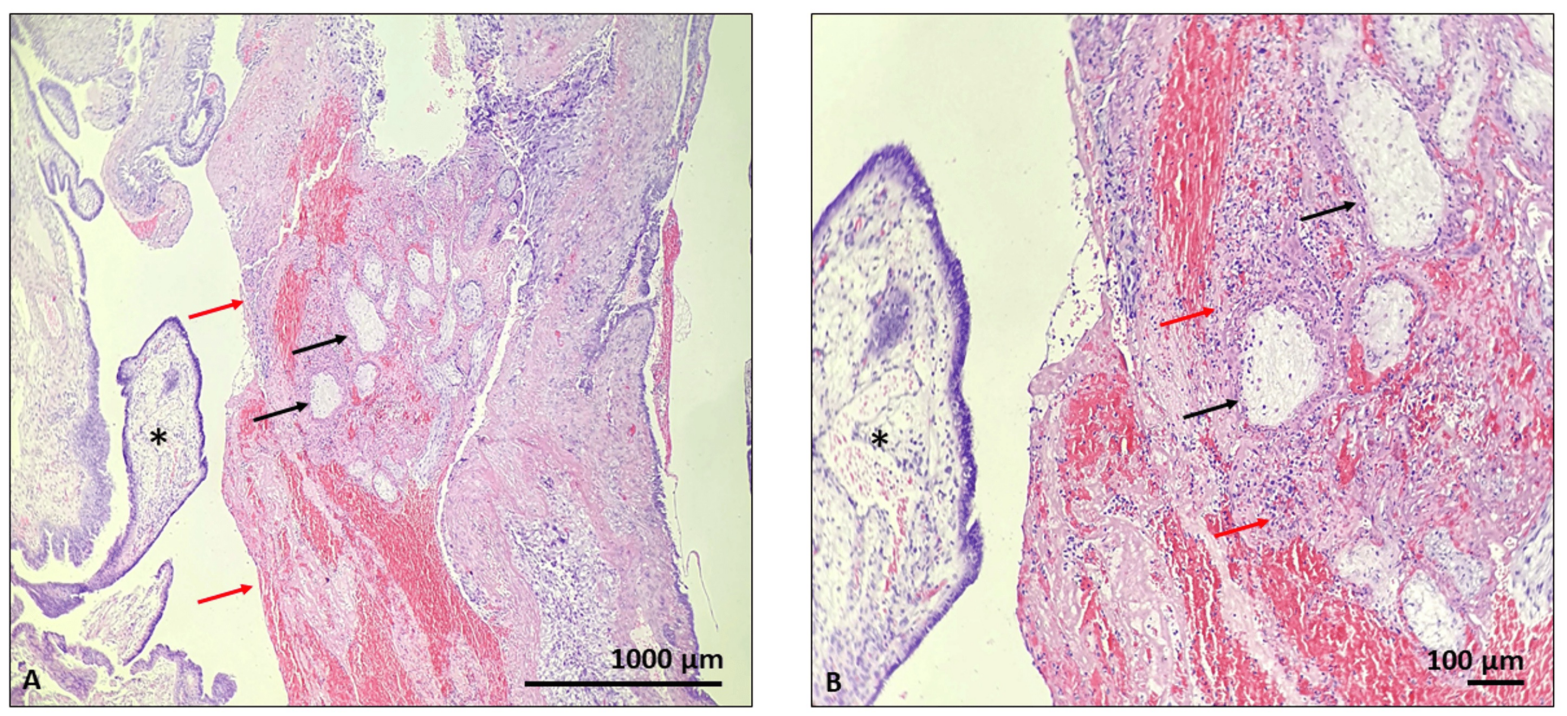
Histological specimen of the ruptured fallopian tube Hematoxylin and eosin staining: visible intraluminal chorionic villi (black arrow), mixed with blood cells, fibrin and cell debris (red arrow), mucosal folds with tubal epithelia (black stars); magnification x40 (A) and x100 (B)

To investigate the cause of the abdominal pain and the ultrasound findings, a diagnostic laparoscopy was performed after informed consent acquisition from the patient. A laparoscopic technique was employed following the administration of general anesthesia. The operative procedure revealed a ruptured right fallopian tube. Due to the extent of the damage, a right salpingectomy was performed as the fallopian tube could not be salvaged. For the histological examination, hematoxylin and eosin staining and visualized the intraluminal chorionic villi with trophoblasts within fibrin and mucosal folds with tubal epithelia. The histological sample confirmed ectopic pregnancy in the fallopian tube, which had caused the fallopian tube to rupture (Figures 3A, 3B, 4A, 4B). 

Postoperatively, the patient recovered well, was monitored regularly, and experienced no further complications. The intrauterine pregnancy remained undisturbed during the laparoscopy, allowing it to progress normally. Follow-up fetal scans were routinely performed to monitor the development of the fetus, which showed a normally developing fetus. The patient later delivered vaginally at 38 weeks gestation.

## Discussion

Heterotopic pregnancies occur in approximately one in 30,000 pregnancies conceived naturally. With assisted reproductive techniques, the incidence increases up to one in 1,500 [1]. The rarity of this condition results in fewer cases being encountered in clinical practice, which often leads to a lack of familiarity among physicians regarding its presentation and management. Consequently, the condition may frequently be overlooked and fail to be included in the differential diagnosis. Due to the potential consequences of late discovery, including maternal mortality, it is crucial to identify heterotopic pregnancy expeditiously. This can be done by educating physicians and other healthcare professionals who are involved in the care of pregnant women on the signs and symptoms of the condition, and management.

Heterotopic pregnancy must be regarded as a differential diagnosis in pregnant women exhibiting relevant symptoms; however, this condition may also occur in individuals lacking significant risk factors, including a history of IVF, tubal surgery, previous ectopic pregnancies, or post-conception levonorgestrel usage. A comprehensive sonographic evaluation is crucial for patients presenting with stomach discomfort or vaginal bleeding to exclude extrauterine pregnancies, even when an intrauterine pregnancy is identified, regardless of the presence of established risk factors.

Moreover, the presence of a normal intrauterine pregnancy in heterotopic pregnancy often delays diagnosis, as it shifts clinical focus toward the normal pregnancy and away from investigating other potential causes of symptoms. Since the intrauterine pregnancy is typically healthy, further diagnostic testing is not routinely performed, which can result in a missed diagnosis. Early detection is essential to prevent complications such as tubal rupture, internal hemorrhage, and death. Women presenting with signs and symptoms of heterotopic pregnancy, which includes, abdominal pain, adnexal mass, peritoneal irritation, or an enlarged uterus, should be thoroughly evaluated [4]. Also, women who have risk factors for the condition such as conception using ART should routinely be examined to rule out the condition. Transvaginal ultrasound is the diagnostic modality of choice to rule out a heterotopic pregnancy. On ultrasound, the extrauterine pregnancy may appear as a mass, distention of the fallopian tubes, or fluid in the pouch of Douglas [9].

## Conclusions

Heterotopic pregnancy should be considered as a differential diagnosis in patients presenting with abdominal pain. The presence of an intrauterine gestational sac does not exclude the possibility of an ectopic pregnancy and thorough examinations should be routinely performed to prevent grave complications and death. Increasing education and awareness among healthcare workers about the clinical presentation of heterotopic pregnancy is crucial for improving the recognition and management of the condition.
